# Lyophilized Chitosan/xanthan Polyelectrolyte Complex Based Mucoadhesive Inserts for Nasal Delivery of Promethazine Hydrochloride 

**Published:** 2014

**Authors:** Mohamed Hassan G Dehghan, Marzuka Marzuka

**Affiliations:** a *Department of Pharmaceutics, Maulana Azad Educational Trust’s, Y. B. Chavan College of Pharmacy, Dr. Rafiq Zakaria Campus, Rauza Bagh, Aurangabad-431001(M.S.), India.*; b *Department of Pharmaceutics, Maulana Azad Educational Trust’s, Y. B. Chavan College of Pharmacy, Dr. Rafiq Zakaria Campus, Rauza Bagh, Aurangabad-431001(M.S.), India.*

**Keywords:** Promethazine hydrochloride, Polyelectrolyte complexes, Lyophilized mucoadhesive inserts, *In*-*vitro* drug release, *Ex*-*vivo* permeation

## Abstract

The objective of this investigation was the development of chitosan/xanthan polyelectrolyte complex based mucoadhesive nasal insert of promethazine hydrochloride a drug used in the treatment of motion sickness. A 3^2^ factorial design was applied for preparing chitosan/xanthan polyelectrolyte complex and to study the effect of independent variables *i.e*. concentration of xanthan [X_1_] and concentration of chitosan [X_2_] on various responses *i.e. *viscosity of polyelectrolyte complex solution, water uptake of nasal inserts (at pH 2, 5.5, 7.4), bioadhesion potential of nasal inserts and *in-vitro *drug release at Q_6h_ through nasal inserts. FTIR and DSC analysis were carried out to confirm complex formation and on loaded and unloaded nasal insert to investigate any drug excipient interaction. The nasal inserts were also characterized by powder X-ray diffractometry (PXRD) and Scanning electron microscopy (SEM) and for *ex-vivo *permeation studies. The results show that higher amount of xanthan in polyelectrolyte complexes with respect to higher amount of chitosan retarded *in-vitro *drug release. The water uptake behaviour of nasal insert was strongly influenced by pH of the medium and by polycation/ polyanion concentration. The investigation verifies the formation of polyelectrolyte complexes formation between chitosan and xanthan at pH values in the vicinity of pKa intervals of the two polymers and confirms their potential for the nasal delivery of promethazine hydrochloride.

## Introduction

Intranasal (IN) administration represents a viable option for local and systemic delivery of diverse therapeutic compounds (1). The large absorptive surface area and high vascularity of the nasal mucosa ensure a rapid onset of therapeutic effect; potential for direct-to-central nervous system delivery and circumvention of the hepatic first pass elimination has made IN delivery a potential alternative among mucosal sites for systemic delivery of drugs with poor oral bioavailability (2). Moreover, IN delivery is non-invasive, essentially painless, does not require sterile preparation, and the easy accessibility facilitates self-medication thus improves patient compliance when compared to parenteral routes. Promethazine hydrochloride {(RS)-dimethyl [1-methyl-2-(phenothiazin-10-yl) ethyl] amine hydrochloride} has antiemetic, antivertigo, anti-motion sickness, anticholinergic effects and local anestheticactions (3). It is used commonly in the treatment of motion sickness. The drug is available on the market in injectable, oral and rectal dosage forms (3). IV and IM routes provide 100% and 75% bioavailability respectively, but are invasive routes and IM injection causes considerable irritation at the site of injection, while IV administration of promethazine possess risk of severe tissue injury, including gangrene requiring amputation (4). While low oral (25%) and rectal bioavailability (23%) of the drug has been attributed to its extensive hepatic first-pass metabolism (5). As intranasal route is noninvasive and is able to attain high bioavailability, there is a need to administer promethazine hydrochloride intra-nasally.

A major problem with nasal delivery is the mucociliary clearance, which rapidly removes applied dosage forms from the absorption site (6). Generally, conventional nasal formulations like nasal drops or sprays are rapidly cleared from the nasal cavity and residence times in man of 10-15 min has been described (7). Although the residence time of a liquid vehicle can be increased by increasing its viscosity, viscous solutions are difficult to administer as drops or sprays. Powder formulations have been shown to have longer nasal residence times (8) than solutions but require sophisticated delivery devices for deposition and accurate dosing. Recently, freeze-drying technology has been applied to the manufacture of unit dose, fast dissolving dosage forms, such as rapidly disintegrating tablets, wound dressings, and ocular drug delivery systems. This approach has also been suggested for the preparation of nasal inserts (9) and nasal inserts with prolonged drug delivery (10).

The purpose of this study was the preparation and characterization of lyophilized nasal inserts so as to enable delivery of a unit dose of drug in the nasal cavity and achieve a sustained release of the active principle.

Xanthan gum, is an anionic microbial exopolysaccharide consisting of a cellulosic backbone, namely β-(1,4)-d-glucopyranoseglucan, with a trisaccharide side chain, namely (3,1)-α-d-mannopyranose-(2,1)-β-d-glucuronic acid-(4,1)-β-d-mannopyranose, on every second glucose residue. Chitosan, is a cationic polysaccharide consisting mainly of poly-β-(1- 4)-d-glucosamine. When chitosan is cross-linked or complexed with an oppositely charged polyelectrolyte, a three-dimensional network is formed in which the drug can be incorporated to control its release (11). These polymers show interesting biological properties, including biocompatibility, biodegradability and mucoadhesivity. Thus in this study chitosan/xanthan polyelectrolyte complexes (PEC) were used to prepare mucoadhesive nasal inserts, these inserts form a gelled system on hydration. The use of chitosan/xanthan PEC gels as carriers has been reported in literature (12- 15). 

A suspension of chitosan/xanthan complexes, with or without promethazine hydrochloride, was lyophilized into small inserts. Morphological characteristics, water uptake, mucoadhesion, release and permeation studies were performed in order to investigate the potential of the insert for nasal delivery of promethazine hydrochloride.

## Experimental

*Materials*

The drug promethazine hydrochloride was procured as gift sample from Wockhardt Ltd, Aurangabad (M.S.) India. Xanthan gum (Molecular weight 2x10^6^ dalton approximately), chitosan (Molecular weight between 110,000-150,000 dalton and degree of de-acetylation 92%) and mannitol were purchased from Research fine Lab. Mumbai, India. All other chemicals and reagents used were of Analytical Reagent Grade. Cellulose acetate membrane (pore size 0.22 μm) was procured from Millipore, Bangalore. Goat (*Capra aegagrushircus) *nasal mucosa was procured from local slaughter house, Aurangabad.

*Preparation of chitosan/xanthan complexes*

A 3^2^ factorial design (Table 1) was applied to prepare chitosan/ xanthan polyelectrolyte complexes and to study its effect on evaluation parameters. The advantages of a factorial design include the greater precision that can be obtained in estimating the overall main factor effects and investigation of the interactions between different factors. By using a factorial design, it is possible to examine the effect of one variable when other factors are changed, something which is not possible using traditional methods of investigation. Briefly chitosan was dissolved in 100 mL of acetate buffer pH 5.0. Xanthan gum was dissolved in 100 mL of distilled water. Both the solutions where mixed together and stirred at room temperature for 24 h. The precipitates were separated by ultracentrifugation at 10,000 rpm for 10 min, washed with deionised water and finally dried under vacuum to constant weight (16).

**Table 1:** Variables and their levels for factorial design

**Variables**	**Levels**		
	**Lower (-1)**	**Middle (0)**	**Upper (+1)**
X_1_-Concentration of xanthan gum (Xa)	0.6% w/v	1 % w/v	1.4 % w/v
X_2_-Concentration of chitosan (CS)	0.6% w/v	1 % w/v	1.4 % w/v

*Manufacture of nasal insert*

The dried complexes were homogenized at 4000 rpm for 15 min, washed with deionized water and then suspended in deionized water. Drug and mannitol were separately dissolved in about one third of the required amount of deionized water. To the homogenized dispersed complexes, drug mannitol solution was added to obtain loaded inserts. The resultant suspension were then filled in polypropylene micro-centrifuge tubes (V=1.5 mL), allowed to swell and any entrapped air was removed and finally lyophilized for 24 h in a freeze drier; with pre-set cycle stages: freezing (4 h, temperature at -30 ^0^C), drying for 20 h, with vacuum 50 mTorr and condenser temperature at -50 ^0^C. The unloaded inserts were prepared by the same procedure without the presence of drug. All inserts had a cone like shape (average diameter of 7 mm, height 8 mm), the average weight of the loaded inserts were 70.2 ± 0.147 mg. The inserts were stored in a desiccator until use. Mannitol was added as bulking agent in order to improve mechanical strength of lyophilized nasal inserts during handling. The amount of polymer complex for drug loaded nasal inserts was 35 mg while mannitol (10 mg) and drug (25 mg) were kept constant for all the batches or runs (Table 2).

**Table 2:** Batches for unloaded and loaded Nasal inserts

**No. of runs code**	**Formulation code**		**X**_1_	**X**_2_
	**For unloaded nasal insert**	**For loaded nasal insert**		
1	C1	MC1	-1	-1
2	C2	MC2	0	-1
3	C3	MC3	+1	-1
4	C4	MC4	-1	0
5	C5	MC5	0	0
6	C6	MC6	+1	0
7	C7	MC7	-1	+1
8	C8	MC8	0	+1
9	C9	MC9	+1	+1

*Viscosity and pH of the polyelectrolyte complex dispersion*

The complexes formed were re-dispersed in deionised water homogenized at 4000 rpm for 15 min. The viscosity of the resulting dispersion was determined at 25 ^0^C ± 1 ^0^C using a Brookfield viscometer DV-II LV (Spindle No. 64). The pH of the dispersion prior to freeze drying was measured using a digital pH meter.

*Drug content*

Drug content of the nasal inserts were determined using U V spectroscopic method in 0.01 M HCl at 249 nm. The drug content was calculated taking 910 as the value for A. (1%, 1 cm) adapted from the method given in the Indian Pharmacopoeia 2007 for promethazine injections (17).

*Water uptake*

Accurately weighed drug loaded inserts were placed on filter paper (40 mm in diameter) soaked in different media (pH 2.0, pH 5.5 and pH 7.4 phosphate buffers) and positioned on top of a sponge (5 cm x 5 cm x 2 cm) previously soaked in the hydration medium and placed in a petri dish filled with the same buffer to a height of 0.5 cm (16). Water uptake was determined, as increase in weight of the insert after 6 h, using the equation given below: 

% Water Uptake (%WU) = (W_Hip_ -W_Hp_ –W_Di_) X 100 / W_Di_

Where, W_Hip_ is the weight of hydrated insert and wet filter paper, W_Hp_ is the weight of wet filter paper; W_Di_ is the initial weight of the dry insert.

*Bioadhesion potential of insert*

One hundred grams of hot agar solution (1 %w/w, in phosphate buffer pH 5.5,) was cast on a petri plate and left to gel at 4–8 ^0^C for 3 h. The gel was then equilibrated for 1 h. to the test conditions of 22 ^0^C and 79% relative humidity in a chamber (18).The inserts which were placed on top of the gel, moved downward due to gravity after the glass plate was turned into a vertical position. The displacement in cm was measured as a function of time (*n=*3). The adhesion potential was inversely related to the displacement of the insert.

*In-vitro drug release studies*

A locally fabricated diffusion cell as reported by Werner U. (19) was used for drug release studies mimicking the humidity properties of the nasal mucosa. The lower end of polypropylene tube having inner diameter of 3.5 cm was placed over the donor compartment having a surface area of 7.07 cm^2^. The receptor compartment was separated from the donor compartment with the help of a cellulose acetate membrane (Millipore 0.22 μm pore size). The receptor compartment was filled with 50 mL phosphate buffer (PBS) pH 5.5 and adjusted exactly to the height of the release medium surface so that the cellulose acetate membrane was wetted but not submersed. Briefly, the receptor compartment contained PBS, pH 5.5 at 37 ^0^C, and the donor compartment contained air saturated with moisture generated by the temperature and the closed system nature of the experimental setup. The nasal insert was placed on cellulose acetate membrane (Millipore 0.22 μm pore size) maintained just in contact with the liquid phase of the receptor compartment, which was constantly agitated with a magnetic stirrer. Samples of 1 mL were withdrawn at regular time intervals from the receptor compartment and analyzed spectrophotometrically (UV-1800, Shimadzu Corporation, Kyoto, Japan.) at 249 nm. Each sample taken from the receptor compartment was replaced immediately with 1 mL of fresh medium.

*Ex-vivo drug permeation studies*

Locally fabricated diffusion cell as reported by Werner U (19), was used for the permeation test. The diffusion chamber with an exposed tissue surface was filled with 50 mL phosphate buffer (pH 5.5). The excised nasal mucosal membrane was secured over the mouth of the upper tube keeping the mucus side exposed to the nasal insert. The nasal insert was placed on mucosal surface of goat nasal mucosa maintained just in contact with the liquid phase of the receptor compartment, which was constantly agitated with a magnetic stirrer. Samples of 1 mL were withdrawn at regular time intervals from the receptor compartment and analyzed at 249 nm (UV-1800, Shimadzu Corporation, Kyoto, Japan). Each sample taken from the receptor compartment was replaced immediately with 1 mL of fresh medium. The cumulative promethazine hydrochloride permeated per unit area was plotted against time and the slope of the linear portion of the plot was taken as the steady state flux (Jss). The permeability coefficient (Kp) under steady-state conditions across excised mucosa has been mathematically expressed, as follows;

Kp = Jss/ Cv

Where, Jss is the steady state flux of concentration in steady state and Cv is the total donor concentration of the formulation concentration in donor.

*Kinetic analysis of in-vitro drug release data*

From the drug release data, the best fit models for each formulation were determined by using the software PCP DISSO V3.

*Scanning electron microscopy*

Inserts were cut with a razor blade to expose the inner structure, fixed on a sample holder with double-sided tape and coated with gold under an argon atmosphere using a gold sputter module in a high vacuum evaporator to a thickness of 6.5 nm (16). The samples were then observed with a scanning electron microscope using secondary electron imaging at 5 kV in order to examine the surface morphology and structure of the insert.

*FTIR analysis*

Fourier transform infrared spectroscopy in the range 400-4000 cm^-1^ was recorded on powder samples of drug, polymers, complex, C1 and MC1 using FTIR−4100 spectrophotometer (resolution 4 cm^-1^; Jasco Corporation, Japan).

*DSC analysis*

DSC analysis was performed for drug, polymers, complex, C1 and MC1 formulation using a DSC instrument (Shimadzu TA 60WS). Each sample was accurately weighed (~1-3 mg) in an aluminum pan, crimped, and hermetically sealed, while an empty pan of the same type was used as a reference. The system was calibrated with high purity sample of indium. The samples were scanned at the heating rate of 20^ o^C/min over a temperature range of 80 ^o^C to 280 ^o^C under the nitrogen atmosphere (19).

*Powder X-ray diffraction*

Powder X-ray diffraction patterns were measured in order to evaluate the crystalline/amorphous character ofuntreated drug, physical mixture and inserts prepared by freeze drying. Measurements were performed using a Philips X-ray generator PW 1830 equipped with a copper anode (40 kV, 30 mA) coupled to a computer-interfaced diffractometer control unit (XPERT-PRO). The scattered radiation was measured with a vertical goniometer (PW 3050/60) (19).

*Stability studies*

Stability studies of the best formulation was done as per to ICH guidelines. The formulation was kept in a stability chamber (Thermo lab, Mumbai, India.) for a period of three months at temperature 40 ^0^C ± 2 ^0^C and RH 75% ± 5%. The changes in physical appearance, weight, drug content, *in-vitro *drug release was observed after intervals of one month.

*Multiple regression analysis of 32 factorial batches*

The responses obtained from 3^2^ factorial batches were subjected to multiple regression analysis. The polynomial equations (20) were determined using the form

Yi= b_0_+b_1_X_1_+b_2_X_2_+ b_11_X_1_^2^+ b_22_X_2_^2^ + b_12_X_1_X_2_+ b_12_ X_1_ X_2_^2^+ b_12_ X_1_^2^ X_2_+ b_12_ X_1_^2^ X_2_^2^

Where Yi is the dependent variable, b_0_ is the arithmetic mean response of the 9 runs, and b_1_ is the estimated coefficient for the factor X_1_. The main effects (X_1_ and X_2_) represents the average results of changing one factor at a time from its low to high value.

The term X_1_^2^ and X_2_^2^ indicate curvilinear relationship. The interaction X_1_X_2_ shows how the dependent variable changes when two or more factors are simultaneously changed. The targeted response parameters were statistically analyzed by applying one-way analysis of variance (ANOVA) at 0.05 levels in Design-Expert 7.1.6 version software (Stat-Ease Inc., Minneapolis, MN).

## Results and Discussion

*Viscosity measurement of polyelectrolyte complex solution and pH measurement of suspension*

**Figure 1:**
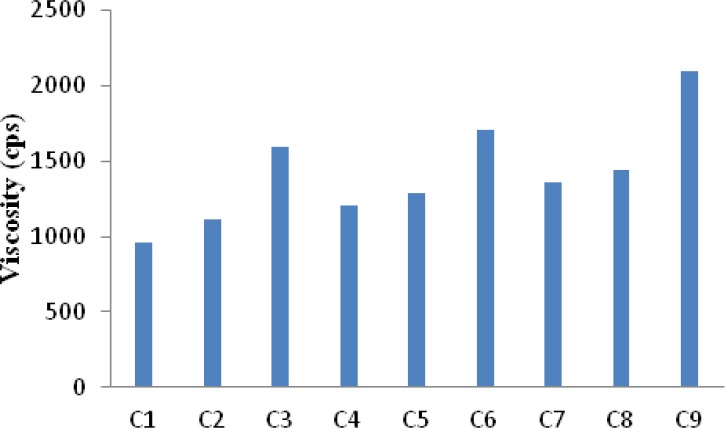
Viscosity of chitosan/ xanthan complex solution (at 25 °C ± 1°C, n=3).

The viscosity of all the formulation batches was determined by measuring the viscosity of polyelectrolyte complex solution. The results (Figure 1) indicated that C1 showed a least viscosity of 960 cps whereas C9 showed the highest viscosity of 2100 cps. Higher amount of xanthan in the complexes provide for the formation of a three dimensional hydrogel structure which is responsible for higher viscosity. It is known that the normal physiological pH of nasal mucosa is between 4.5 and 6.5. To avoid nasal irritation, the pH of the nasal formulation should be adjusted to 4.5 - 6.5. At this pH in addition to avoiding irritation, it results in obtaining efficient drug permeation and prevents the growth of bacteria (21). The pH of gel solutions were measured and it was found to be within the range 5.5 to 5.8 (Table 3).

*Drug content*

Determinations of the total drug content of individual nasal inserts are shown in Table 3. The total drug content exhibits the drug loading for single nasal insert. The drug content was found to be uniform in all the batches thus indicates complete drug loading of nasal inserts.

**Table 3:** Viscosity, pH measurement and drug content of Nasal insert (±SD, n=3).

**Formulation code**	**Drug content(±SD)n=3**	**pH(cps) (±SD) n=3**	**Viscosity (±SD) n=3**
MC1	99.64 ± 0.64	5.51 ± 0.1154	960 ± 7
MC2	99.40 ± 0.37	5.66 ± 0.1154	1115 ± 3.055
MC3	99.51 ± 0.95	5.57 ± 0.0577	1590 ± 9.848
MC4	98.53 ± 0.72	5.78 ± 0.0577	1210 ± 7.767
MC5	99.09 ± 0.95	5.56 ± 0.0577	1290 ± 7.549
MC6	98.98 ± 0.80	5.54 ± 0.0577	1710 ± 6.506
MC7	99.85 ± 1.40	5.6 ± 0.1154	1360 ± 8.3266
MC8	99.69 ± 1.14	5.8 ± 0.1154	1440 ± 9.643
MC9	99.65 ± 0.2	5.71 ± 0.0577	2100 ± 8.3266

*Water uptake*

**Figure 2:**
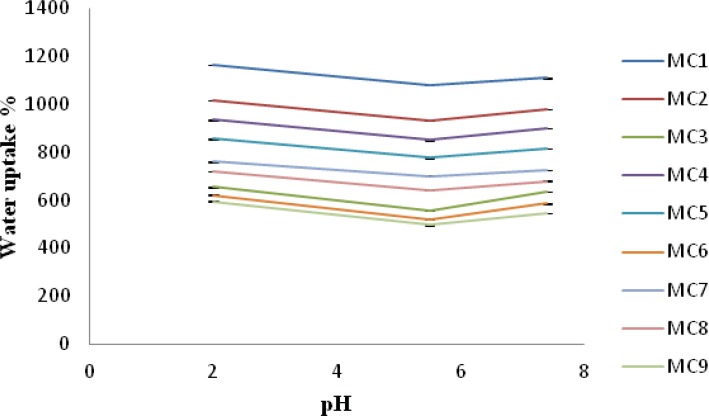
Water uptake (%) of Nasal insert at pH 2, 5.5 and 7.4 (mean ± SD) n=3 after 6 h.

Water uptake ability of chitosan/xanthan polyelectrolyte complexes was strongly influenced by pH of the medium and by polycation /polyanion concentration during the formation of complex. As can be seen in Figure 2, water uptake ability was lower at pH 5.5 than at pH 7.4 for all the batches analyzed. Water uptake ability was found to be higher at pH 2 than compared to pH 7.4 and pH 5.5. In fact, when complexes hydrated in the pKa interval of the two polysaccharides, the interactions between negative and positive charges in the polymeric network underwent only little or no modification, resulting in a lower water uptake. On the contrary, a large excess of free positive or negative charges appears inside the polymeric network at pH 2 and 7.4, thus allowing greater water uptake. Among all the formulations MC1 showed highest water uptake ability at pH 2, 7.4 and 5.5 whereas formulation MC9 showed least water uptake ability at pH 2, 7.4 and 5.5. This indicates that a complete crosslinking of both the polymers occurs in case of MC8, MC3, MC6 and MC9 formulation where the concentration of xanthan gum was higher *i.e*. 1%-1.4% w/v and so the water uptake was lower for these batches. While in case of MC1 crosslinking density was low hence higher water uptake was found. This result corroborates well with those reported by Soysal A S, *et al. *(13) Moreover the presence of promethazine hydrochloride in the nasal insert gradually reduced water uptake. This behavior can be explained due to the presence of the amino group (pKa 9.2) of promethazine (22) which is able to interact with free negative charges (xanthan carboxylate groups) in the complex during the loading procedure, thus leading to formation of less porous inserts (18).

*Bioadhesion potential of insert*

**Figure 3:**
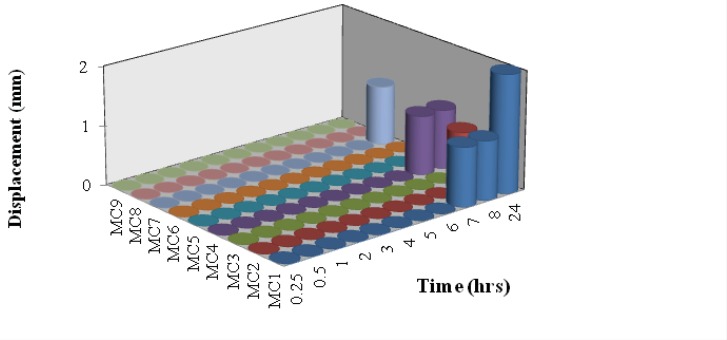
Bioadhesion potential of Nasal insert (n=3).

The vertical displacement of inserts on an agar plate was used as a measure of bioadhesion potential. The adhesion potential is inversely related to the displacement of the insert (18). After administration into the nasal cavity and contact with the moist surface, freeze dried insert hydration produces gelling network able to interact with mucus as a result of physical entanglement and secondary binding. In fact all batches showed good bioadhesion potential (Figure 3). MC1 showed displacement only after a period of 6 h. In case of MC2 it showed displacement at 8 h. MC4 showed displacement above 8 h whereas MC7 showed displacement at 24 h. While all the other batches showed zero displacement even after a study period of 24 h. This may be due to increase in concentration of xanthan gum in complexes of following batches MC3, MC8, MC9, MC5 and MC6. At pH 5.5, mucus presents negative charge due to complete ionization of sialic acid (pKa 2.6) and sulphate residues in mucin glycoprotein (23). Despite the presence of negative charge on xanthan chains due to ionization of the carboxyl groups, xanthan showed good mucoadhesive potential. On the other hand, despite the presence of positive charges on chitosan chains due to the ionization of the amino groups, chitosan shows lower mucoadhesive ability.

*In-vitro drug release*

**Figure 4:**
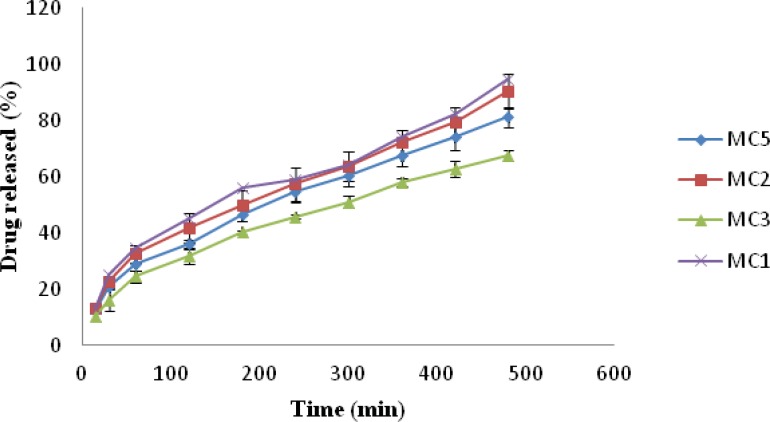
Plot of Drug release vs. Time of formulation MC1, MC2, MC3 and MC5

**Figure 5:**
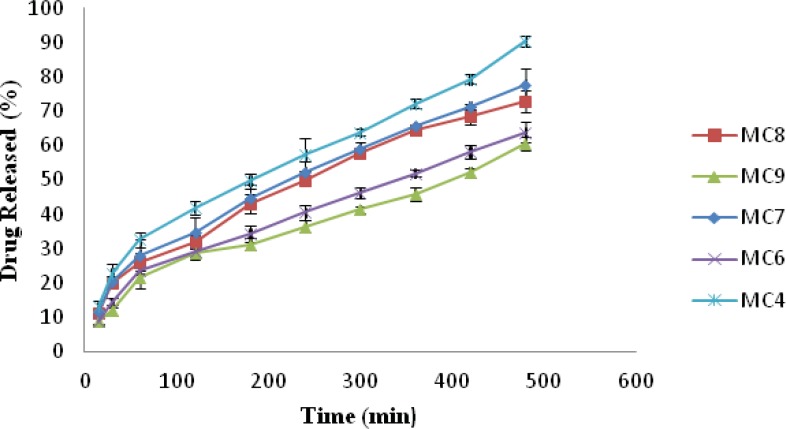
Plot of Drug release vs. Time of formulation MC4, MC6, MC7, MC8 and MC9

The release of drug from nasal inserts is a complex phenomenon of water penetration, relaxation of the polymer chains, swelling and spreading of the insert, dissolution of the water soluble polymer and drug, interactions of the drug and carrier, and drug diffusion through the rehydrated insert (18). From the drug release vs. time profile Figure 4, it is evident that amongst all the batches MC1 (0.6% w/v of chitosan and xanthan gum) showed highest release of 94.93% followed by MC2 (1% w/v of xanthan gum and 0.6% w/v of chitosan) 90.36% release. MC9 [1.4%w/v chitosan and xanthan gum] formulation showed lowest drug release *i.e*. 60.67%, Figure 5. The release for a period of up to 6 h is studied taking into consideration the limited nasal residence due to eventual mucocillary clearance. These results correlate well with the results obtained for viscosity and water uptake for these batches. Thus there exists an inverse relationship between viscosity and drug release, the apparent viscosity/micro-viscosity of the formulation influence the diffusion of the particles, when the characteristic length is larger than the length scale of the structure elements in the formulation. In case of MC1 due to lower viscosity and higher water uptake at pH 5.5 release of drug was faster when compared to other batches. The probable reason for this can be due to the low degree of cross linking density between chitosan and xanthan in the complex and presence of free charges which allow higher water uptake mobility and thus higher release rate. While in case of batch MC9 due to complete crosslinking density and absence of free charges limiting water uptake and polymeric chain mobility may be the reason for lower release of drug from MC9.

*Ex-vivo permeation studies*

**Figure 6:**
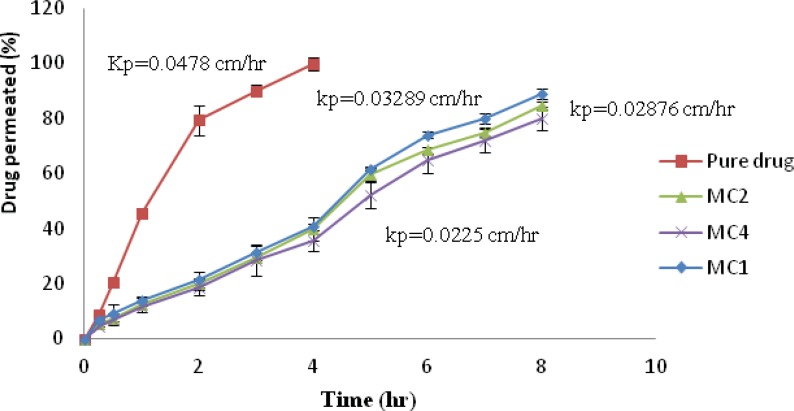
Plot of percentage of Drug permeated vs. Time for pure drug and formulation MC1, MC2, MC4.

Formulations, showing higher *in-vitro *drug release with good bioadhesiveness, were selected to study permeation through nasal mucosa. The *ex-vivo *permeation for aqueous drug solution and formulations MC1, MC2 and MC4 through nasal mucosa were determined, Figure 6. It was observed that the permeation of pure drug from aqueous solution (25 mg/mL) shows 99.86% within 4 h, whereas formulation MC1, MC2, MC4 showed 89.016%, 84.88%, and 79.87% after 8 h respectively. The permeation of promethazine from nasal insert formulations was found to be low as compared with aqueous drug solution. Pure drug solution showed higher flux (Jss) and permeability coefficient (Kp) than formulation MC1, MC2 and MC4. Jss and Kp for pure drug solution was 1.196 mg/cm2/h. and 0.04785 cm/h. respectively, while among the formulations MC1 showed highest flux and permeability coefficient of 0.8223 mg/cm2/h. and 0.03289 cm/h. respectively. ANOVA followed by Dunnett multiple comparison test revealed statistically significant difference when the batches were compared with pure drug solution whereas among the batches MC1, MC2 and MC3 no significant difference was observed (p < 0.05).

*Kinetic analysis of in-vitro drug release data*

As observed from the *in-vitro *drug release kinetic data, formulation MC1, MC2, MC3, MC4, MC5, MC6, MC7, MC8, MC9, MC10 show Higuchi matrix type of release as best fit model (Table 4). The n-values are more than 0.5, which indicates non-Fickian release *i.e. *initially there is a rapid release, followed by tailing off over time.

*Scanning electron microscopy*

The structure of the nasal insert depends on the composition of chitosan/xanthan complexes.

**Figure 7:**
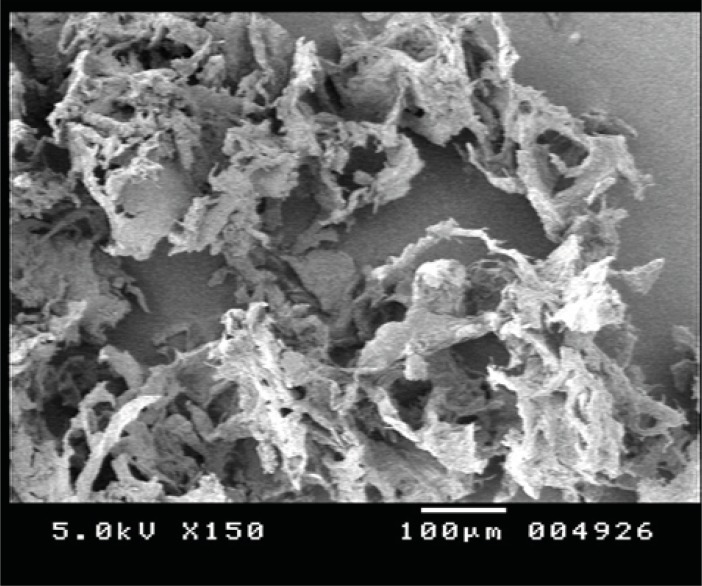
Scanning electron microscopy of formulation MC1

For polyelectrolyte complexes, the interaction of polycation with polyanion leads to physically crosslinkedhydrogels (15) that can retain great amount of water at the interior. As nasal inserts were obtained by freeze drying, which consists of sublimation of the frozen water yielding to the formation of pores or channels in the polymer, all the inserts were characterized by sponge-like structure this is seen in the SEM of the nasal insert formulation MC in Figure 7.

*FTIR analysis*

**Figure 8:**
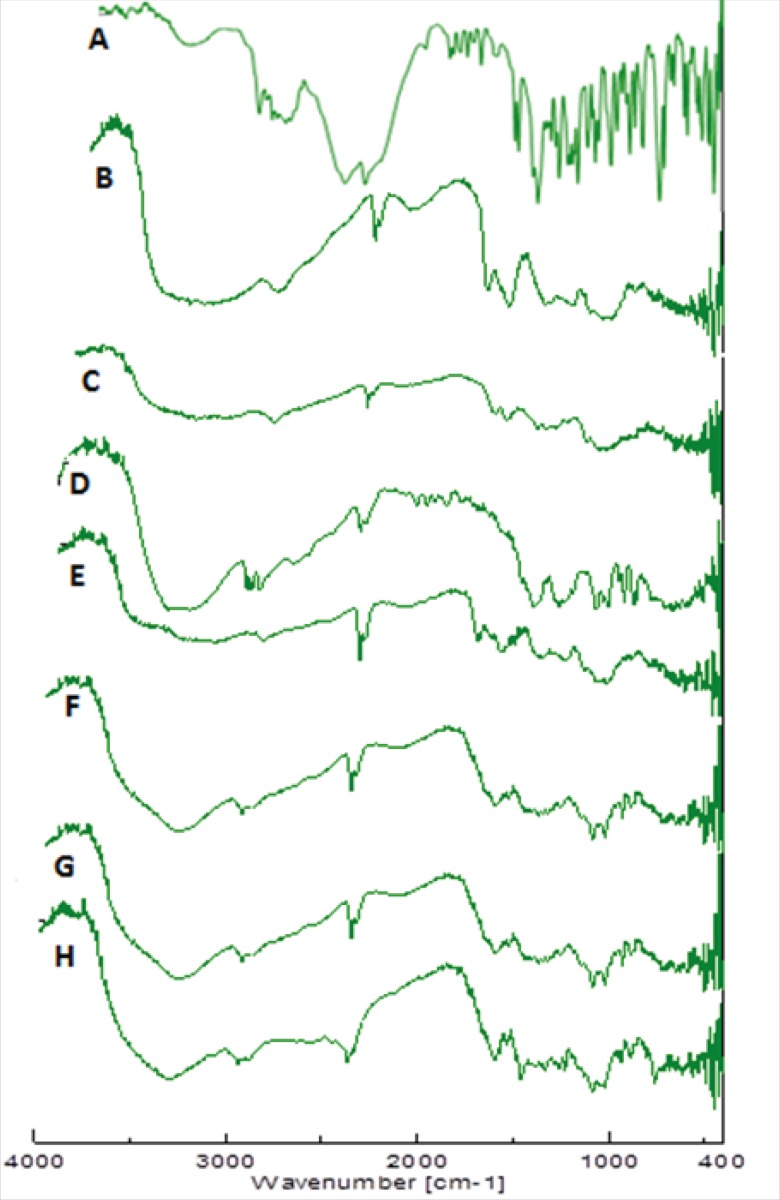
FTIR Spectra of promethazine hydrochloride (A), xanthan gum (B), chitosan (C), mannitol (D),chitosan/xanthan polyelectrolyte complex (E), physical mixture (F), unloaded nasal insert-C1(G) ,Drug loaded nasal insert formulation MC1(H).

FTIR of chitosan/xanthan polyelectrolyte complex Figure 8(E) confirmed the formation of complex between chitosan and xanthan gum. FTIR spectra of xanthan gum Figure 8(B) showed typical ν_c=o_ band of carboxylate at 1620 cm^-1^, whereas chitosan Figure 8(C) showed the characteristic ν_c=o_ band of amide at 1648 cm^-1^ and δ_N-H_ band of amine at 1584cm^-1^. The complex showed δ_N-H_ band characteristic of protonated amine at 1529 cm^-1^, FTIR spectra of physical mixture Figure 8(F) showed clearly the characteristic peaks of complex, drug and mannitol. FTIR spectra of unloaded insert (C1) and loaded insert of formulation MC1 Figure 8 (G) and Figure 8(H) were also taken to note any changes that occur during freeze drying. The spectrum of unloaded inserts show characteristic peaks of complex as well as of mannitol, whereas the FTIR spectra of loaded inserts and physical mixture show dominant peaks of drug molecule, but intensity weakens due to physical interaction between complex and drug molecule. It may be due to weak ionic interaction between them.

**Table 4:** Release Kinetics of *in-vitro *drug release

**Formu-lation code**	**R value**					**Best fit model**	**Parameters for Korsemeyer Peppas equation**		
	**Zero** **order**	**First** **order**	**Matrix**	**Peppas**	**Hixson ** **Crowell**		** k**	**n**	
MC1	0.8600	0.9652	**0.9917**	0.9878	0.9556	**Matrix**	30.5856	0.5060	
MC2	0.8803	0.9738	**0.9951**	0.9925	0.9623	**Matrix**	28.7609	0.5092	
MC3	0.8842	0.9649	**0.9991**	0.9980	0.9445	**Matrix**	22.0880	0.5102	
MC4	0.8966	0.9854	**0.9979**	0.9943	0.9700	**Matrix**	27.3489		0.5114
MC5	0.8897	0.9818	**0.9985**	0.9952	0.9630	**Matrix**	26.4265		0.5128
MC6	0.9013	0.9679	**0.9972**	0.9950	0.9513	**Matrix**	19.8559		0.5142
MC7	0.8883	0.9787	**0.9984**	0.9942	0.9588	**Matrix**	25.4663		0.5168
MC8	0.8911	0.9753	**0.9978**	0.9938	0.9555	**Matrix**	24.3509		0.5198
MC9	0.8886	0.9586	**0.9879**	0.9932	0.9434	**Matrix**	18.1774		0.5260

*DSC analysis*

**Figure 9:**
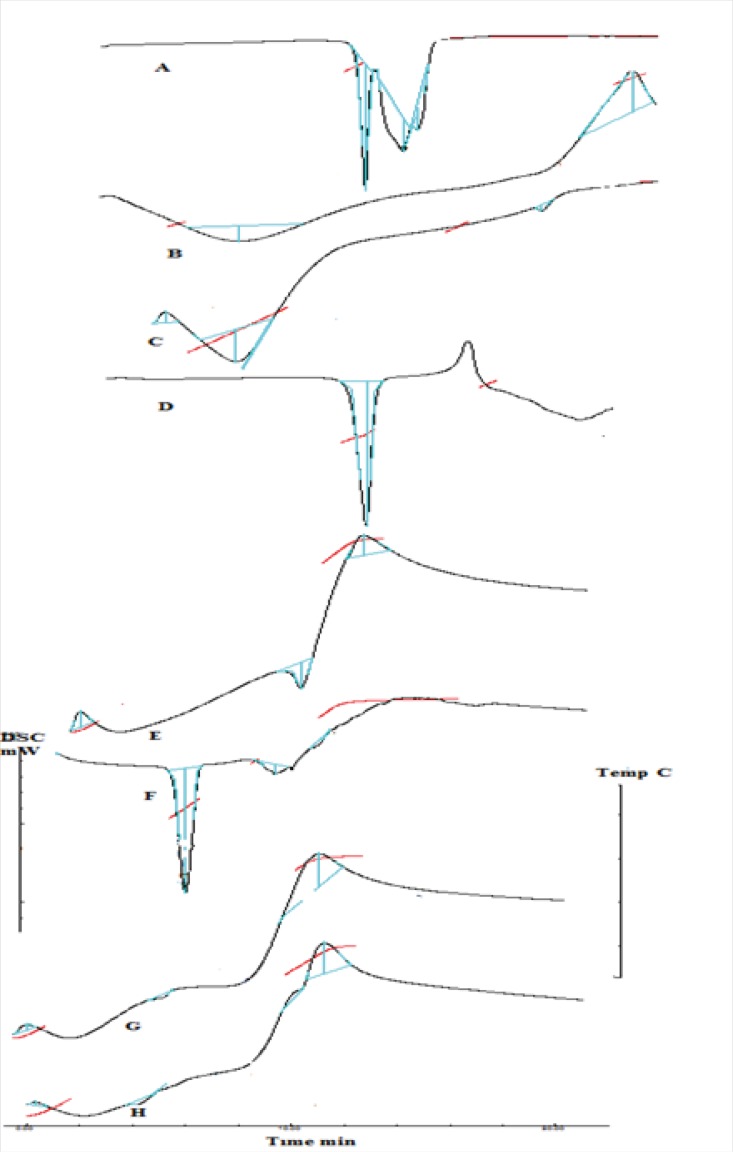
DSC thermogram of promethazine hydrochloride (A), xanthan gum (B), chitosan (C), mannitol (D),chitosan/xanthan polyelectrolyte complex (E), physical mixture (F), unloaded nasal insert-C1(G) ,Drug loaded nasal insert formulation -MC1 (H).

DSC thermogram of drug, polymers, complex, physical mixture and formulation C1 and MC1 were obtained. Promethazine hydrochloride Figure 9(A), shows a characteristic endothermic peak at 238.96 °C which corresponds to its decomposition melt. DSC thermogram of xanthan gum, Figure 9(B) and chitosan Figure 9(C) showed a glass transition temperature characterized by a change in heat capacity, which is seen as a change in the baseline (24), peak at 116.10 °C and 108.42 °C respectively. DSC thermogram of mannitol Figure 9(D) showed a characteristic peak of 172.24 °C which indicates its melting point. The thermogram of the complex Figure 9(E) showed an endotherm with peak at 228.57 °C, the disappearance of Tg seen for chitosan and xanthan gum is indicative of the complex formation. The thermogram of physical mixture Figure 9(F) showed an endotherm at 221.92 °C corresponding to promethazine hydrochloride. The difference in thermal peaks between the pure components and physical mixture blend may be attributed to sample geometry effects and to reduction of individual purity in the presence of other component (24). DSC thermogram of unloaded nasal insert showed exothermic peak at 267.22 °C corresponding to the exothermic peak of complex seen at 267.81 °C Figure 9(G) and an endothermic peak at 171.57 ^0^C corresponds to mannitol. The thermogram of loaded inserts Figure 9(H) showed an endothermic peak with onset at 233.32 °C and peak at 248.43 °C corresponding to the melting point of the drug.

*PXRD analysis*

**Figure 10:**
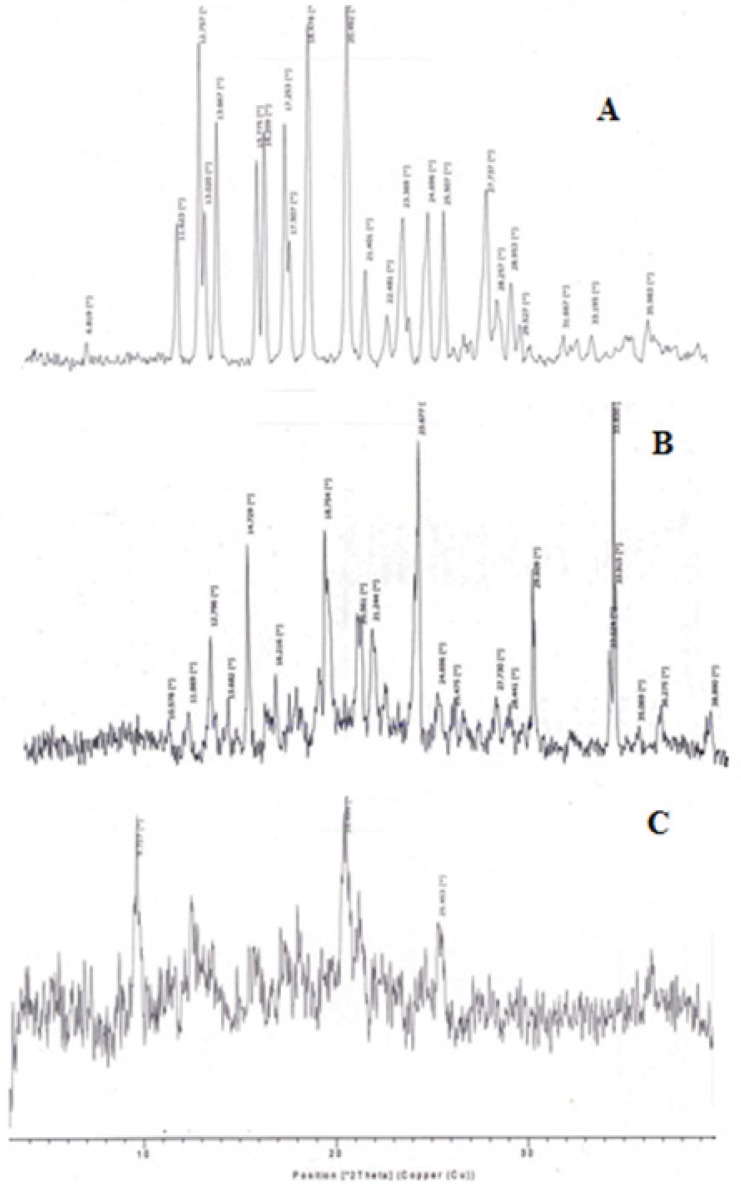
PXRD of Promethazine Hydrochloride (A), Physical mixture [(complex: drug: mannitol) 1:1:1], (B), drug loaded nasal insert formulation MC1(C).

PXRD analysis of the drug was performed to confirm its crystalline structure. The diffraction pattern of promethazine Figure 10(A), showed maximum intensity peak at [°2θ] value equal to 20.492, other sharp peaks at [°2θ] values 18.478, 12.757, 13.66, 17.53, 27.737, 24.696, 16.209, 21.401 were noticeable. The diffraction pattern of physical mixture, Figure 10(B), was also highly crystalline in nature as indicated by numerous peaks. Sharp peaks at [°2θ] value equal to 23.677, 33.830, 18.754, 14.729, 29.609, 12.796 were observed. The diffraction pattern of freeze dried formulation MC1 (loaded insert) showed reduction in sharp peaks, Figure 10(C), thus it indicates a resultant amorphous state of mixture due to lyophilization.

*Stability studies*

Batch MC1 was subjected to stability studies for a period of three months (40 °C ± 2 °C and 75% ± 5% RH). The stability data of formulation MC1 is presented in Table 5. Physical appearance of the nasal inserts was same as initial condition. The drug content of the inserts after storage for 3 month was within limits. Weight of nasal inserts increased when compared to initial weight, it may be due to moisture uptake from the storage environment. *In-vitro *release of promethazine hydrochloride was observed to be highest from the insert after a time interval of 3 month; this may be due to increased hydration as a result of moisture uptake at 75% ± 5% RH by the lyophilized insert.

**Table 5:** Stability evaluation of Nasal insert (40 °C ± 2 °C and 75% ± 5% RH).

**Parameter**	**Initial**	**1 month**	**2 month**	**3 month**
Appearance	Off-white	Off-white	Off-white	Off-white
Weight	70.25 mg	76.50mg	82.75mg	88.54mg
Drug content	99.06%	99.18%	98.56%	97.85%
Microbial growth	Nil	Nil	Nil	Nil

*Multiple regression analysis of 3* ^2^ *factorial batches*

Table 6 A and 6 B shows the statistical evaluation and multiple regression analysis of 3^2 ^factorial batches for six responses along with their derived factorial equation. The RSM, Figure 12, obtained for the relationship between independent variables and the responses Y_1_, Y_2_, Y_3_, Y_4_, Y_5_ and Y_6_ support and substantiate earlier discussions. The surface plot for the response Y_1_ (viscosity) indicates that viscosity increased as both the independent variables increased. Response surface plots for Y_2_ (water uptake at pH 2), Y_3_ (water uptake at pH 5.5), Y_4_ (water uptake at pH 7.4) respectively indicates that water uptake is dependent on both the independent variables, combined effect X_1_X_2_ and X_1_^2^. Water uptake thus decreased with increase in concentration of both xanthan and chitosan. The surface response plot Y_5_ (bioadhesion potential) which shows that the bioadhesion potential increased with increase in concentration of both xanthan and chitosan. Response surface plot for *in-vitro *drug release at Q_6hr _shows that drug release is dependent on both the independent variables, combined effect X_1_X_2_ and X_1_^2^. *In-vitro *drug release at Q_6hr_ thus decreased with increase in concentration of both xanthan and chitosan. The effect of the independent variables on all the responses chosen for the study is imperative considering the relationship between viscosity, bioadhession potential, water^1^ uptake and *in-vitro *drug release from the insert.

**Figure 11:**
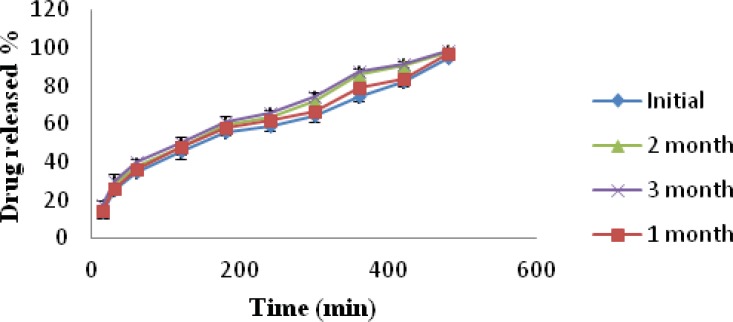
Plot of % Drug released vs. Time profile of initial, 1 month, 2 month, 3 month stability study formulation MC1.

**Figure 12:**
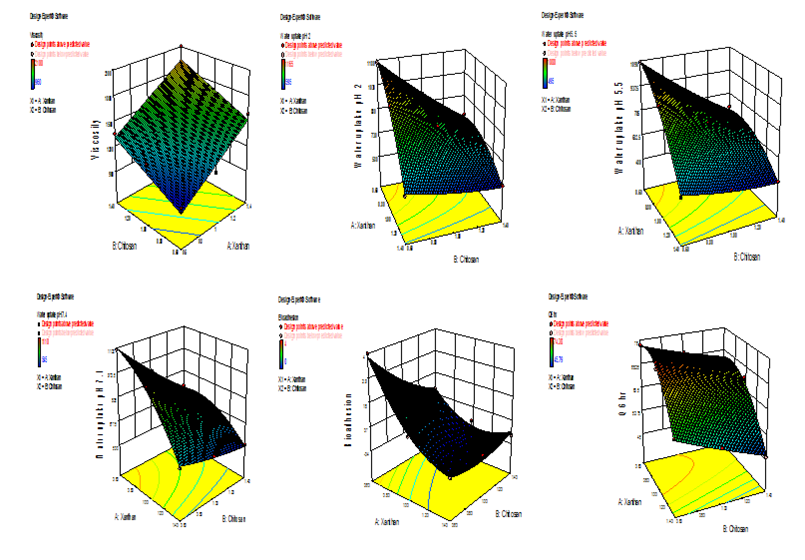
Response Surface Plot showing the effect of variables on; Viscosity of polyelectrolyte complex solution (Upper Left).

Water uptake of Nasal insert at pH 2 (Upper Middle).

Water uptake of Nasal insert at pH 5.5 (Upper Right).

Water uptake of nasal insert at pH 7.4 9 (Lower Left).

Bioadhesion potential of nasal inserts (Lower Middle).

*In-vitro *drug release at Q_6h_ from nasal insert (Lower Right).

*Multiple regression analysis of 3* ^2^ *factorial batches*

Table 6 A and 6 B shows the statistical evaluation and multiple regression analysis of 32 factorial batches for six responses along with their derived factorial equation. The RSM, Figure 12, obtained for the relationship between independent variables and the responses Y_1_, Y_2_, Y_3_, Y_4_, Y_5_ and Y_6_ support and substantiate earlier discussions. The surface plot for the response Y_1_ (viscosity) indicates that viscosity increased as both the independent variables increased. Response surface plots for Y_2_ (water uptake at pH 2), Y_3 _(water uptake at pH 5.5), Y_4_ (water uptake at pH 7.4) respectively indicates that water uptake is dependent on both the independent variables, combined effect X_1_X_2_ and X_1_^2^. Water uptake thus decreased with increase in concentration of both xanthan and chitosan. The surface response plot Y_5_ (bioadhesion potential) which shows that the bioadhesion potential increased with increase in concentration of both xanthan and chitosan. Response surface plot for *in-vitro *drug release at Q_6hr_ shows that drug release is dependent on both the independent variables, combined effect X_1_X_2_ and X_1_^2^. *In-vitro *drug release at Q_6hr_ thus decreased with increase in concentration of both xanthan and chitosan. The effect of the independent variables on all the responses chosen for the study is imperative considering the relationship between viscosity, bioadhession potential, water^1^ uptake and *in-vitro *drug release from the insert.

**Table 6:** (A). Multiple regression analysis of 3^2^ factorial batches for viscosity, bioadhesion potential and Q_6h_

**Source**		**Degree of freedom**	**Sum square**			**Mean square**		**F-value**			**Prob>F**		
**Y**_1_ **= Viscosity**													
Model		2	8.370E+005			4.185E+005		23.25			0.0015		
X_1 _		1	5.828E+005			5.828E+005		32.38			0.0013		
X_2_		1	2.542E+005			2.542E+005		14.14			0.0094		
R^2^=0.887 Adj R^2^=0.8476 PredR^2^=0.7416 SD=134.16 CV=9.45													
**Equation Y**_1_ **=1419.44+311.67 X**_1_ **+205.83 X**_2_													
**Y**_5_ **=Bioadhesion potential**													
Model	5		14.69		2.94				45.34			0.0050	
X_1_	1		8.17		8.17				126.00			0.0015	
X_2_	1		2.67		2.67				41.14			0.0077	
X_1_X_2_	1		2.25		2.25				34.71			0.0098	
X_1_^2^	1		1.39		1.39				21.43			0.0190	
X_2_^2^	1		0.22		0.22				3.43			0.1612	
R^2^=0.986 Adj R^2^=0.9652 Pred R^2^=0.8467 SD=0.25 CV=2.24													
**Equation Y**_5_ **= 0.11-1.17X**_1_ **-0.67X**_2_ ** +0.75X**_1_ ** X**_2_ ** +0.83X**_1_^2^ **+0.33 X**_2_^2^													
**Y**_6_ **=** ***In-vitro *** **drug release at Q**_6h_													
Model	5			727.34			145.47			144.56			0.0009
X_1_	1			482.57			482.57			479.57			0.0002
X_2_	1			139.50			139.50			138.63			0.0013
X_1_X_2_	1			3.13			3.13			3.11			0.1759
X_1_^2^	1			101.92			101.92			101.28			0.0021
X_2_^2^	1			0.22			0.22			0.22			0.6721
R^2^=0.9959 Adj R^2^=0.9890 Pred R^2^=0.9513 SD=1.00 CV=1.58													
**Equation Y**_6_ **= 67.86-8.97X**_1_ **- 4.82X**_2_ **-0.88X**_1_ ** X**_2_ **-7.14X**_1_^2^ **+0.33 X**_2_^2^													

(* Significant terms at P< 0.05).

**Table 6:** B Multiple regression analysis of 3^2^ factorial batches for water uptake at pH 2, 5.5 and 7.4.

**Source**			**Degree of freedom**				**Sum square**	**Mean square**	**F-value**	**Prob>F**
**Y**_2_ **=Water uptake at pH 2**										
Model				5			3.011E + 005	60214.58	122.30	0.0012
X_1_				1			1.634E + 005	1.634E+005	331.72	0.0004
X_2_				1			96266.67	96266.67	195.52	0.0008
X_1_X_2_				1			29756.25	29756.25	60.64	0.0044
X_1_^2^				1			11250	11250	22.85	0.0174
X_2_^2^				1			450	450	0.91	0.4096
R^2^=0.9951 Adj R^2^=0.9870 Pred R^2^=0.9406 SD=22.19 CV=2.73										
**Equation Y**_2_ **=853.33-165.00 X**_1_ **-126.67 X**_2_ **+86.25 X**_1_ **X**_2_ **-75.00 X**_1_^2^ **+15.00 X**_2_^2^										
**Y**_3_ **=Water uptake at pH 5.5**										
Model		5			3.15E + 0056			3138.89	89.72	0.0018
X_1_		1			1.873E + 005			1.873E+005	266.12	0.0005
X_2_		1			88816.67			88816.67	126.21	0.0015
X_1_X_2_		1			25600.00			25600.00	36.38	0.0091
X_1_^2^		1			13338.89			13338.89	18.96	0.0224
X_2_^2^		1			672.22			672.22	0.96	0.4005
R^2^=0.9934 Adj R^2^=0.9823 Pred R^2^=0.9201 CV=3.65 SD=26.53										
**Equation Y**_3_ **= 769.44-176.67X**_1_ **-121.67 X**_2_ **+80.00 X**_1_ **X**_2_ **-81.67X**_1_^2^ **+18.33X**_2_^2^										
**Y**_4_ **=Water uptake at pH 7.4**										
Model	5					2.902E + 005		58047.92	128.01	0.0011
X_1_	1					1.568E + 005		1.568E+005	345.81	0.0003
X_2_	1					1.001E + 005		1.001E+005	220.75	0.0007
X_1_X_2_	1					21756.25		21756.25	47.98	0.0062
X_1_^2^	1					11250.00		11250.00	24.81	0.0156
X_2_^2^	1					312.50		312.50	0.69	0.4673
R^2^=0.9953 Adj R^2^=0.9876 Pred R^2^=0.9432 SD=21.29 CV=2.75										
**Equation Y**_4_ **= 816.67-161.67X**_1_ **-129.17X**_2_ ** +73.75X**_1_ **X**_2 _ **-75.00X**_1_^2^ ** +12.50X**_2_^2^										

* (Significant terms at P< 0.05).

## Conclusion

Chitosan/xanthan polyelectrolyte complex mucoadhesive nasal inserts has a good potential for use as a delivery system for promethazine hydrochloride a drug used in treatment of motion sickness. FTIR and DSC analysis confirmed the formation of complex between chitosan/xanthan and also confirms that there was no chemical interaction of the drug with the other components used in the formulation. The release kinetics showed Higuchi matrix type of drug release which obeys a non-Fickian diffusion process. PXRD analysis on nasal inserts indicated conversion of drug and excipients to amorphous form after lyophilization. SEM analysis of nasal inserts showed the formation of porous structure, which is prerequisite for its *in-situ *gelling property. The selection of suitable chitosan/xanthan concentration during complex preparation allowed modulation of insert water uptake behaviour and promethazine hydrochloride release and permeation at the administration site. Finally, formulation MC1 was the best formulation as it showed good *in-vitro *drug release (94.93%), water uptake as well as bioadhesive characteristics with nasal mucosal permeation of 89.018% in 8 h.
